# Ethnic and Gender Disparities in Premature Adult Mortality in Belize 2008-2010

**DOI:** 10.1371/journal.pone.0163172

**Published:** 2016-09-19

**Authors:** Francis Morey, Ian R. Hambleton, Nigel Unwin, T. Alafia Samuels

**Affiliations:** 1 Department of Health Services, Ministry of Health, Belmopan, Belize; 2 Chronic Disease Research Centre, Caribbean Institute for Health Research, The University of the West Indies, Bridgetown, Barbados; 3 Chronic Disease Research Centre, Caribbean Institute for Health Research, The University of the West Indies, Bridgetown, Barbados; National Institute of Health, ITALY

## Abstract

**Background:**

Data on disparities in mortality *within* low and middle income countries are limited, with little published data from the Caribbean or Central America. Our aim was to investigate disparities in overall and cause specific premature adult mortality in the multi-ethnic middle income country of Belize.

**Methods:**

Mortality data from Belize 2008–2010 classified using the International Classification of Diseases 10 and the 2010 census stratified by age and ethnicity were used to calculate age, sex, and ethnic specific mortality rates for those 15–59 years, and life table analysis was used to estimate the probability of death between the ages of 15 and 59 (*45q15*).

**Results:**

The probability of death among those aged 15 to 59 years was 18.1% (women 13.5%, men 22.7%). Creole and Garifuna ethnic groups have three times the *45q15* probability of death compared to Mayan and Mestizo groups (Creole 31.2%, Garifuna 31.1%, Mayan 10.2%, Mestizo 12.0%). This pattern of ethnic disparity existed in both sexes but was greater in men. The probability of death from injuries was 14.8% among Creole men, more than twice the rate of other ethnicities and peaks among young Creole men. These deaths are dominated by homicides and unspecified deaths involving firearms

**Conclusions:**

Marked disparities in mortality between ethnic groups exist in this Central American/Caribbean country, from rates that are typical of high-income countries to those of low-income countries. The pattern of these extreme differences likely suggests that they reflect underlying social determinants rooted in the country’s colonial past.

## Introduction

The region of the Americas has undergone a fundamental demographic and health transition, characterized by decreased communicable disease mortality, lower fertility, and population aging[1]. This public health success creates new healthcare challenges, with an increasing burden of chronic disease. In the 40 years between 1965–70 and 2005–10, life expectancy at birth in the Americas has increased by 14.6 years to 73.5 years; 9.7 years (to 72.7 years) in the Caribbean, 16.5 years (to 75.3 years) in Central America and 13.9 years (to 73.1 years) in South America. However, the overarching regional improvement masks within-region disparities in human longevity and country-level disparities also exist. In the Caribbean, 40-year life expectancy improvements ranged from 4.5 years to 15.9 years, and life expectancy at birth in 2005–10 ranged from 60.7 to 80.1 years[2].

In the region, and especially in the Caribbean, little is known about within-country disparities in life expectancy and mortality. Health disparities are widely linked to the concept of unfair health and healthcare access among population subgroups due to gender, race/ethnicity, socioeconomic status, or other socially determined factors. In 2005, the World Health Organization (WHO) established the Commission on the Social Determinants of Health[3]. That report proposed 3-actions: improve the conditions of daily life, tackle the inequitable distribution of resources globally, nationally, and locally, and measure the problem, evaluate action, and expand the knowledge-base[4].

This report focuses on Belize; the only English-speaking country in Central America, bordered by Mexico, Guatemala and the Caribbean Sea. A former British colony, its history and politics are similar to the English Speaking Caribbean[5], and it is a full member of the Caribbean Community (CARICOM)[6]. Belize is classified as a upper-middle income country with a Gross National Income per capita (2010) of US$4,180[7] and a 2010 population of 323,359[8]. Life expectancy at birth since 1965–70 has increased by 8.4 years, reaching 72.7 years in 2005–10 (increased by 10.2 years to 75.6 years among women, by 6.8 years to 69.9 years among men). Median age is 21 years and 52% of the population live in urban areas. In 2005, adult literacy was 94.7% among both males and females, although only 58.7% of eligible children went on to secondary school[9].

Belize is one of the most ethnically diverse countries of the Americas. Almost half (49%) of the population are Mestizo, 21% are Creole, 10% are Maya, and 5% are Afro Amerindian (Garifuna), with smaller numbers of European, East Indian, Chinese, Middle Eastern, and North American groups[8].

The Belizean Mestizo people are of mixed Maya and European descent. They are geographically and economically diverse, spread fairly evenly in 4 districts, including the coastal Corozal district, with high unemployment and high poverty rates; inland in Cayo district with high unemployment; and in Orange Walk and Belize districts[9, 10].

The Creole people are of mixed African and European descent, with the term Creole identifying a culture rather than a physical appearance. Two thirds of Creoles live in Belize district, which includes Belize city, the largest city in the country.

The Maya are the indigenous people of Belize and the Yucatán regions. About 60% live in Toledo, one of the two poorest districts, located in the south of the country.

The Garifuna are a mix of African, Arawak, and Carib ancestry. Half live in coastal Stann Creek, south of Belize district and one third live in the Belize district. Dandriga, the principal town of Stann Creek district, is now a favorite tourism culture destination, where there Afro-Caribbean traditions can be shared[10].

Health inequities in Belize are associated with urbanization, gender and ethnicity. The rural poverty rate of 55% is double that of the urban rate of 28% and has been increasing. Unemployment among women is double the rate among men[9]. The Global Burden of Disease profile for Belize[11] indicates that between 1990 and 2010, premature mortality (assessed as years of life lost, YLL) from interpersonal violence increased by 1,692%, rising from 29^th^ rank to 5^th^ rank within Belize. Self-harm increased by 401% from 26^th^ rank to 12^th^ rank and drug use disorders increased by 307%. HIV/AIDS increased by 721% and diabetes by 288%.

Belize has begun its epidemiological and demographic transition. Despite the increase in HIV/AIDS, there have been declines in most other Group 1 diseases–Communicable, maternal, neonatal and nutritional (GBD). Population growth has slowed. Between 1991 and 2010 the 0–4 years population declined from 16% to 12% of the total[9].

Drawing on the recommendations from the WHO Commission on Social Determinants of Health[4], our rationale is to explore within-country mortality differentials in Belize by gender and ethnicity, using sex-stratified national mortality statistics including recorded ethnicity of the deceased[8]. Our main focus is on premature adult mortality, defined as mortality between the ages of 15 and 59 years, given its acknowledged importance as a determinant of economic and social development[12]. The within-country mortality differentials we report provide an expanded evidence-base to aid public health policy as there are very few published works reporting ethnic disparities in total mortality and underlying causes among English-speaking Caribbean populations living in the Caribbean[13–17].

The aims of this study, therefore, were to determine if differences exist in premature adult mortality between the four main ethnic groups in Belize, by gender, possible conditions underlying such differences, and to identify implications for reducing health inequities.

## Materials and Methods

This research was approved by the Institutional Research Board of the Ministry of Health/University of the West Indies, Barbados and Ministry of Health, Belize.

### Data Sources

This analysis uses data on deaths that occurred between 2008 and 2010 which included date of death, underlying cause of death, and sex and ethnicity of the deceased [18]. Deaths data is collated by the Vital Statistics Unit of Belize, with cause of death statistics generated by the Ministry of Health Epidemiology Unit, who use this mortality information as a basic metric for tracking national health trends[19]. National mortality statistics are submitted annually to the Pan-American Health Organization (PAHO), who maintain a regional mortality database from 40 countries and territories in the Americas[20]. This regional database does not stratify deaths by ethnicity. Data on the size of the Belize population, stratified by age, sex, and ethnicity from the 2010 population and housing census[8], were provided by the Statistical Institute of Belize and is available on-line at http://www.sib.org.bz/statistics/population

This data is also in supplemental files in S1 File, refer to tables 22–25.

### Mortality Groups

Underlying cause of death in Belize between 2008 and 2010 was verified by the Belize Epidemiology Unit, and classified using the International Classification of Diseases (ICD, 10^th^ edition). We further classified underlying cause of death into three broad groups: communicable diseases, non-communicable diseases, and injuries (intentional and unintentional). This broad cause of death grouping has been used by the World Health Organization and others when reporting regional and country-level mortality statistics[21]. We stratified the broad injury grouping into smaller cause of death groups to explore reasons for these deaths in greater detail. The groups used were: homicides and deaths involving firearms (ICD10 X93-95), suicides (ICD10 X60-84, Y87), road traffic accidents (ICD10 V01-89), accidents (ICD V01-X59, Y85-86), and other causes of injury (ICD10 X85-92, X96-99, Y00-Y10, Y35-36, Y40-98).

### Statistical Methods

We constructed period life tables by ethnicity and by sex, to describe the survival pattern of our population group across broad age ranges, given the age-specific death rates observed between 2008 and 2010[22]. This methodology does not make allowance for likely future changes in mortality, making period life expectancy somewhat ‘historical’ in nature. Nonetheless, it is a succinct summary, and provides an objective means of comparing trends in mortality over time. Our life table summary measures included the probability of death between the ages of 15 and 59, based on probability of death by 5 year age groups, commonly known as *45q15*[12]. We tabulated 45q15 for all deaths, and for our three broad (and competing) mortality causes (communicable disease, non-communicable disease, injuries), by ethnicity and gender.

Over the three year study period 50 deaths (1.2%), out of the total of 4,312, were assigned to ill-defined causes (ICD-10 codes R00-R99). These were re-assigned proportionately by age and sex to other causes, apart from injuries.

We investigated the potential overall completeness of the mortality data by estimating the total number deaths that would be expected according to UN life tables[23], which are based on the West variant of the Coale-Demeny model[24], and UN estimates of underlying population size and structure for Belize in 2009[23]. Between 1999 and 2011 the percentage difference between the estimated number of deaths from the UN data and those registered in Belize varied between -8.9% (more deaths registered) and 4.3% (more deaths estimated). Based on this analysis there is no evidence of substantial or consistent under reporting of deaths.

However, another threat to the validity of our analyses is the potential for differential bias that could be introduced by missing data on ethnicity at death registration. Out of the 4,312 registered deaths during the study period, 248 (5.8%) had missing ethnicity data. In our main analyses we re-assigned these to one of 5 ethnic groupings (Mayan, Creole, Garifuna, Mestizo, Other) following the known ethnic distribution from the Belize 2010 census. In sensitivity analyses we explored what difference it would make to our findings if missing data on ethnicity were not missing at random, but was greater in some ethnic groups compared to others. We explored this for changes in *45q15*, as this was the main outcome measure of interest. We assumed for each ethnic group in turn that an additional proportion of the 248 deaths with unknown ethnicity should be assigned to that group. We sequentially assumed additional proportions of 10%, 20%, 30%, 40%, and 50% should be assigned to each ethnic group in turn. We stopped at 50% re-assignment on the grounds that this represents an implausibly extreme scenario. For each additional proportion, and each ethnic group, we undertook 1000 dataset randomizations in order to provide an uncertainty interval (e.g. allowing for the effect of randomly selected differences in age and causes of death in the re-assignment).

We also calculated years of life lost (YLL) until age 65 due to each specific cause of death YLL by ethnic group and sex. YLLs were calculated from the period life tables described above, using the following formula:

PYLLL=x=0LdxL−xm

Where χ is a given age 5-year age interval, *L* is the upper age limit (in this case 65 years), *dx* is the number of deaths occurring in each age interval (calculated from the survivor function), and *x*_*m*_ represents the age interval midpoint i.e. (x + (x + n_x_))/2

We also divided this same YLL statistic by the population size between 15 and 65 years of age, to create a YLL rate (YLLr).

Finally, we calculated directly standardized mortality rates for deaths between 15 and 65 years per 10,000 population, by ethnicity and sex for our three broad mortality causes, including 95% mortality rate confidence intervals calculated using a Gamma approximation, which has improved properties when the numbers of deaths are small[25]. All analyses were performed using Stata statistical software v13 (StataCorp, College Station, TX).

## Results and Discussion

The 2010 Belize population and number of deaths 2008–2010 are presented in Table 1 stratified by ethnicity and sex. There were 4,312 deaths, with age at death and sex of the deceased always reported, and ethnicity reported for 4,064 (94.3%) of the deceased. The population was 323,359 in 2010, of which 194,423 (60.1%) were aged 15 to 65 years. Within this restricted adult age range, there were 1,995 deaths (46.3% of all deaths) resulting in an age-standardized mortality rate between 2008 and 2010 of 34.3 deaths per 10,000 population (24.7 per 10,000 among women and 44.1 per 10,000 among men).

**Table 1:** Population in 2010 and number of deaths between 2008 and 2010 among women and men in Belize.

	Mayan			Creole			Garifuna			Mestizo			ALL †		
	Women	Men	All	Women	Men	All	Women	Men	All	Women	Men	All	Women	Men	All
**Population**															
All ages	15,424	15,439	30,863	34,201	34,050	68,251	7,871	7,250	15,121	79,470	78,469	157,939	161,758	161,601	323,359
0 to 65	14,964	14,846	29,810	32,262	32,381	64,643	7,397	6,914	14,311	76,692	75,321	152,013	155,134	154,641	309,776
15 and older	8,974	9,077	18,051	23,086	22,629	45,715	5,542	4,928	10,470	52,121	50,418	102,538	104,606	103,385	207,991
15 to 65	8,515	8,483	16,998	21,148	20,960	42,108	5,070	4,592	9,662	49,346	47,273	96,620	97,992	96,431	194,423
15 to 60	8,295	8,243	16,538	20,474	20,242	40,716	4,902	4,446	9,349	47,981	45,716	93,697	95,058	93,144	188,202
**No. deaths between 15&65**															
Comm. Diseases	7	6	13	69	106	175	22	42	64	36	54	91	152	236	388
Non-comm. Diseases	32	21	53	153	202	355	36	49	85	180	162	342	467	536	1,003
Injuries	4	31	35	28	220	248	9	17	26	28	178	206	90	514	604
Overall	43	58	101	250	528	778	67	108	175	244	395	639	709	1,286	1,995
**No. deaths between 15&60**															
Comm. Diseases	5	6	11	68	101	169	22	38	60	35	50	85	147	219	366
Non-comm. Diseases	27	17	44	127	156	283	28	39	67	142	132	274	373	421	794
Injuries	4	31	35	28	215	243	7	17	24	27	172	199	86	500	586
Overall	36	54	90	223	472	695	57	94	151	204	354	558	606	1,140	1,746

**†** Total population include other ethnicities (N = 50,256) and people of unknown ethnicity (N = 925).

### Probability of Death: Sex and Ethnic Group Differences

**Fig 1:**
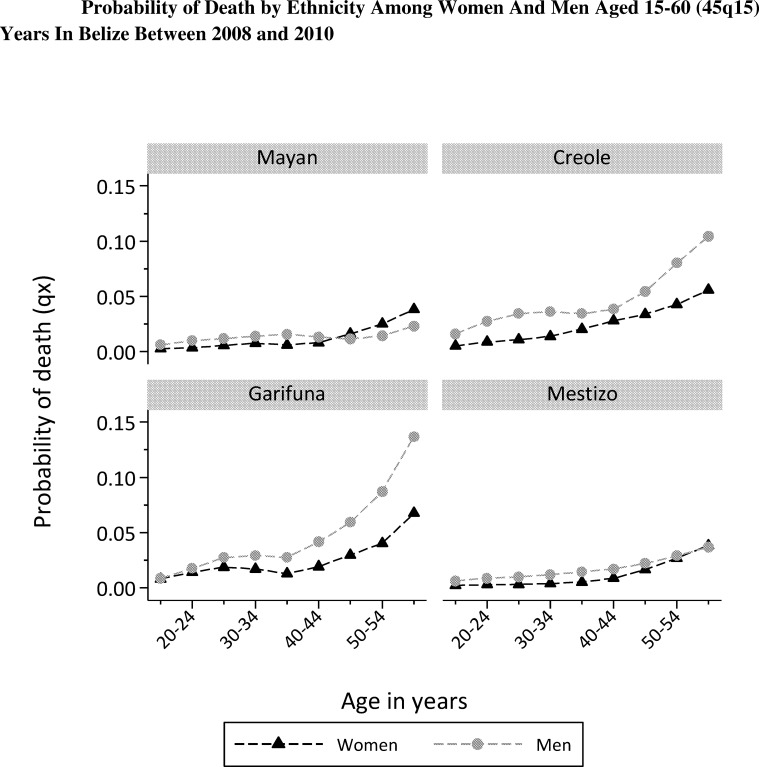
Probability of Death by Ethnicity among Women and Men Aged 15–60 (45q15) Years in Belize Women, Men.

As shown in Table 2, the overall the probability of death among the population of Belize aged 15 to 59 years was 18.2 percent, and was higher in men than in women (men 22.8%, women 13.6%). Important ethnic variation existed, with Creole and Garifuna ethnic groups having three times the 45q15 probability of death compared to Mayan and Mestizo groups (Creole 32.1%, Garifuna 31.8%, Mayan 10.5%, Mestizo 13.1%). This ethnic disparity pattern existed in both sexes but was greater in men (Fig 1).

**Table 2:** Probability of death between 15 and 60 years of age, years of life lost, and age-standardized mortality rate between between 15 and 65 years of age among women and men in Belize (2008–2010).

	Mayan			Creole			Garifuna			Mestizo			ALL *		
	Women	Men	All	Women	Men	All	Women	Men	All	Women	Men	All	Women	Men	All
**Prob of death (45q15) (%)** **‡**															
**Comm. diseases**	1	1.9	1.4	5.3	9.7	7.6	6.3	16.2	11	1.7	2.1	1.9	2.6	4.5	3.6
**Non-comm. Diseases**	8.2	3.5	5.8	14.4	17.4	15.9	13.8	20.1	16.8	8.2	7.4	7.8	9.5	10.4	10
Injuries	0.6	5.9	3.2	2.2	15	8.6	1.7	6.6	4	0.9	5.9	3.4	1.4	7.9	4.6
Overall	9.7	11.3	10.5	21.9	42.1	32.1	21.8	42.9	31.8	10.8	15.5	13.1	13.6	22.8	18.2
**Years of life lost (YLL)** **†**															
Comm. Diseases	1788	2263	4050	4050	5178	9228	1138	1335	2473	5183	5383	10565	13,175	15,173	28,348
Non-comm. Diseases	1185	1095	2280	3368	4160	7528	560	903	1463	4275	4005	8280	10,290	11,703	21,993
Injuries	275	1258	1533	1545	8405	9950	450	713	1163	1428	6993	8420	4,253	19,275	23,528
**Years of life lost Rate (YLLr)** **††**															
Comm. Diseases	39.8	50.8	45.3	41.8	53.3	47.6	51.3	64.4	57.6	22.5	23.8	23.2	28.4	32.8	30.6
Non-comm. Diseases	26.4	24.6	25.5	34.8	42.8	38.8	25.2	43.5	34.1	18.6	17.7	18.2	22.2	25.3	23.7
Injuries	6.1	28.2	17.1	16	86.5	51.3	20.3	34.4	27.1	6.2	30.9	18.5	9.2	41.7	25.4
**Mortality rate** **‡‡**															
Comm. Diseases	2.8	3.5	3.1	11	16.4	13.8	14.4	31.2	22.3	2.8	4.5	3.6	5.2	8.2	6.7
Non-comm. Diseases	15	8.2	11.6	24.9	31.6	28.3	23	34.6	28.4	14.1	12.7	13.4	16.4	18	17.2
Injuries	1.5	12.7	7	4.9	36.1	20.3	6	13.2	9.4	2.1	13	7.4	3.1	17.9	10.4
Overall	19.4	24.4	21.7	40.8	84.1	62.4	43.4	79	60.1	18.9	30.1	24.4	24.7	44.1	34.3

**‡** The probability of death by 60 years of age among people alive at age 15 (45q15)

**†** The potential years of life lost until age 65 due to specific causes of disease. This is a sum of the time lags separating the moment of each death (the midpoint of each age interval) and a conventional upper age limit of 65 years.

**††** YLL expressed as a rate per 1,000 people under 65 years (and so the years of life lost per 1,000 people at risk)

**‡‡** Directly-standardized mortality rate between 15 and 65 years, per 100,000 population aged 15–65

* Total population include other ethnicities (N = 50,256) and people of unknown ethnicity (N = 925).

The probability of death among women in the Creole and Garifuna groups was roughly twice that of the Mayan and Mestizo groups (Creole 21.9%, Garifuna 21.8%, Mayan 9.7%, Mestizo 10.8%). The probability of death among Creole and Garifuna men was between three and four times that of male Mayan and Mestizo men (Creole 42.1%, Garifuna 42.9%, Mayan 11.3%, Mestizo 15.5%) (Table 2).

### Probability of Death: Cause of Death and Ethnic Group Differences

In the Belize population aged 15 to 59 years, the probability of death from non-communicable disease was highest at 10.0%, followed by injuries at 4.6%, and communicable diseases at 3.6%. The same ethnic stratification of higher probability of death among the Creole and Garifuna ethnic groups, and lower probability of death among the Mayan and Mestizo groups persisted for communicable and non-communicable causes of death. Death probabilities for communicable diseases were between 1% and 2.1% among the Mayan and Mestizo groups, and were between 5.3% and 16.2% among the Creole and Garifuna groups. For non-communicable diseases the probabilities were between 3.5% and 8.2% among the Mayan and Mestizo groups, and were between 13.8% and 20.1% among the Creole and Garifuna groups.

### Mortality from Injuries

The probability of death from injuries among the four ethnic groups, ranged from 0.6% to 2.2% among women and 5.9% and 15.0% among men (Table 2).

**Fig 2:**
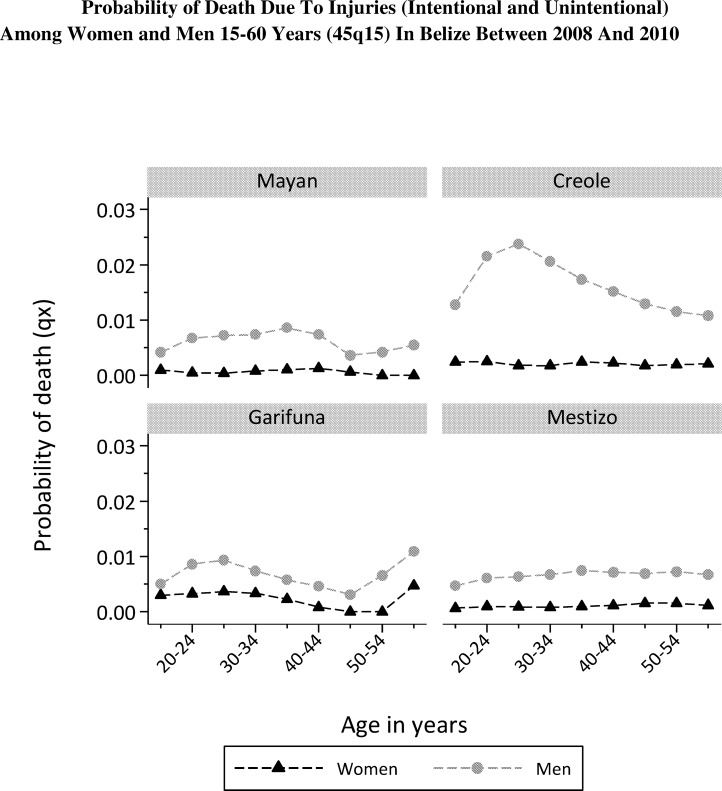
Probability of Death Due To Injuries (Intentional and Unintentional) Among Women and Men 15–60 Years (45q15) In Belize between 2008 And 2010, Women, Men.

The probability of death from injuries was 15.0% among Creole men, more than twice the rate of other ethnicities (Table 2) peaking among young Creole men (Fig 2). Across all ethnicities, these deaths are dominated by homicides and unspecified deaths involving firearms.

### Mortality Rates: Sex, Ethnic Group, and Cause of Death Group Differences

**Fig 3:**
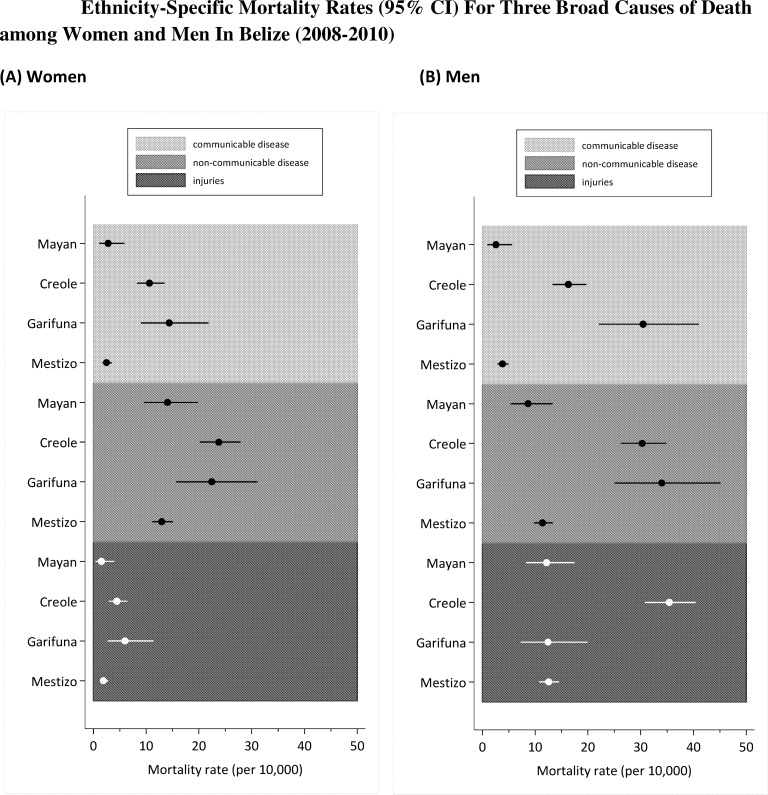
Ethnicity-Specific Mortality Rates or Three Broad Causes of Death among Women and Men in Belize (2008–2010). Mortality rate (per 10,000).

Mortality rates by sex and ethnicity for each cause of death group are presented in Table 2 and Fig 3 (with 95% confidence intervals) for adults between 15 and 65 years of age. Compared to other ethnic groups and other causes of death, Creole and Garifuna females have relatively high rates of non-communicable disease. Among men, there are relatively high rates of injury deaths among the Creole ethnic group, relatively high rates of non-communicable disease deaths among the Creole and Garifuna ethnic groups, and relatively high rates of communicable disease deaths among the Garifuna ethnic group.

### Sensitivity Analyses

As described in the methods, sensitivity analyses were conducted on different approaches to assigning an ethnic group to those deaths where ethnicity was not recorded, and Table 3 shows the effect on overall *45q15*. The first row (0% re-assignment) shows the values on the assumption that ethnicity was missing in proportion to the size of the population of the different ethnic groups, with rows below that showing changes following progressive re-assignment, with up to 50% of deaths of unknown ethnicity being assigned to each ethnic group in turn.

**Table 3:** Minimum to maximum probability of death (45q15) on different levels of assignment of deaths with missing ethnicity: from assignment based on population proportion (0% re-assignment), to 10%, 20%, 30%, 40%, and 50% death re-assignment.

Death reassignment	Mayan min%, max%	Creole min%, max%	Garifuna min%, max%	Mestizo min%, max%
Observed 0% re-assigned	10.5, 12.5	31.8, 32.5	31.3, 32.8	12.8, 13.2
10% re-assigned	11.3, 13.6	32.0, 32.9	32.2, 34.9	12.8, 13.3
20% re-assigned	12.4, 14.2	32.3, 33.3	33.8, 37.0	12.9, 13.4
30% re-assigned	13.1, 15.9	32.6, 33.6	35.3, 38.4	13.0, 13.5
40% re-assigned	14.0, 17.0	32.9, 33.7	36.7, 40.4	13.2, 13.6
50% re-assigned	15.2, 18.3	33.0, 34.2	38.4, 42.0	13.3, 13.6

As would be expected, the findings for the smaller populations (Mayan and Garifuna) are most influenced by re-assigning deaths of unknown ethnicity to those groups. However, the basic pattern of disparities, with markedly higher probabilities of death in the Creole and Garifuna compared to the Mayan and Mestizo persist even under the most extreme re-assignment of deaths. For example, the lowest estimated probability of death in the Creole population is 31.8% compared to a highest probability of 18.3% in the Mayan and 13.6% in the Mestizo. Even under the most extreme re-assignment of deaths the patterns of differences shown in Table 2 persist.

Supporting Information appears in S1 Data.

## Discussion

In this study we have found striking differences in premature adult mortality between the four main ethnic groups in Belize, and between men and women. The probability of death between the ages of 15 and 59 (45q15) in the Mayan and Mestizo, is similar to many richer, economically developed countries of Western Europe and North America[26]. However, when compared to richer parts of the world Mayan and Mestizo men tend to compare more favourably than do women. For example, in the United States[26] the 45q15 in women in 2010 was 7.7% compared to 9.7% and 10% in Mayan and Mestizo women respectively; and men in the US it was 13.0% compared to 11.3% and 15.5% Mayan and Mestizo men.

In contrast the probability of premature adult mortality in the Creole and Garifuna groups is much more like that found in parts of sub-Saharan African and Eastern Europe, particularly in men. For example, in Russia in 2010 the 45q15 for men was 41.2%, very similar to that in Creole (42.1%) and Garifuna (42.9%) men. Similar figures are found in many sub-Saharan African countries[26]. Creole (21.9%) and Garifuna (21.8%) women, however, have a worse 45q15 than described in any Eastern European country (the worst is 15.7% in Russia and Ukraine), but are similar to Senegal (20.1%) and Sudan (20.7%), both of which are have lower female adult mortality than the majority of countries in sub-Saharan Africa.

These conclusions are robust to the sensitivity analyses that we conducted. There is no evidence, for example, of a significant undercount in the total number of deaths. In addition, while ethnicity was not recorded on 5.8% of death registrations, even the extreme assumption that half of these deaths should be assigned to only one of the four ethnic groups had a relatively minor effect on the mortality differences. Our findings are also given credibility by reports of similar levels of disparity within other countries, including the United States. For example, Murray and colleagues defined ‘Eight Americas’, based on county-race combinations, with the most deprived (‘America 8’) being largely made up of urban black populations[27]. In 2001, the 45q15 in America 8 compared to America 1 (the least deprived, predominantly Asian group) was 3.8 and 3.4 times higher in men and women respectively[27]. These differences are similar to what we describe here, with the relative differences in 45q15 between the Creole and Garifuna groups compared to the Mayan being 3.7–3.8 in men, and 2.3 in women.

### Causes of Death Underlying the Differences in Mortality

The mortality data provide some clear pointers as to the conditions underlying these marked differences between ethnic groups in Belize. The probability of death from injuries in Creole men (15.0%) is much higher than among the other three ethnic groups (5.9–6.6%). When years of life lost are examined the relative difference for injuries as a cause of death between male Creole and the other male groups is even greater (Table 2), reflecting the fact that most deaths occur in young men (Fig 2). Approaching half (45.6%) of deaths in Creole men aged 15 to 59 were due to ‘homicide and injury purposively inflicted’.

Injury as a cause of death is much more common in men than women in all groups, with the relative probability (men vs. women) of roughly 4 (in Garifuna) to 8 (in Mayan). Because of this it is easy to overlook the fact that both Creole and Garifuna women also have high rates of injury death compared to women in the other two groups. This is particularly evident when examining rates of years of life lost from injuries, which are roughly 2.5 to 4 times higher in Creole and Garifuna women compared to the other groups. Homicide and transport accidents account for between 50 to 60% of all injury related deaths.

The marked ethnic group differences in death from injury described here are against a background of increasing homicide rates in Belize and its Central American neighbours. Between 2000 and 2009, for example, homicide rates in Belize doubled from 16 to 32 per 100,000 per year[28]. Over the same time period the homicide rates in Guatemala increased from 25 to 50, in Honduras from 50 to 60, and in El Salvador from 35 to 65 per 100,000 per year[28]. The vast majority of homicide victims and perpetrators are men, and the majority of these are young men, who are from poorer urban areas, with high rates of unemployment, poor infra-structure and low levels of educational attainment[29]: the types of areas within which the Creole and Garifuna populations in Belize mainly live. Within such environments gang cultures are rife, typically linked to the illicit drugs trade. The US Government, for example, has identified Belize as among the top 22 illicit drug producing or transit countries in the world[9]. It is estimated that 90% of South American cocaine enters the US via central America[29]. Urban gangs in Belize have been closely linked to gangs in other central American countries and to Mexico[29]. The epi-centre of violence in Belize is Belize district, where roughly two thirds of the Creole and one third of the Garifuna populations live. Belize district contains one third of the population but in a previous study was found to have 55% of the murders, 57% of reported violence against women and 96% of reported child abuse: men at war, the authors noted, also hurt women and children[29]. The origins of the conditions in areas such as Belize district, and the persistent social violence within them, can be linked to the colonial experience and a failure of development following independence, with poorly planned, rapid urbanization without social infrastructure[29]. An additional factor is marked differences in educational expectations and attainment by gender. A study in Jamaica, for example, described how in secondary school, boys are expected to be tough and receive less academic and emotional support from teachers than girls[30]. Boys have been described as, ‘being below the radar, unseen, unnoticed and unattended’[31]. These different gender expectations contribute to a vicious circle of absent fathers due to death, incarceration or migration, and a lack of positive male role models.

Finally, in addition to higher mortality from injuries in Creole and Garifuna groups, mortality is also higher in these groups, men and women, from both communicable and non-communicable diseases (Fig 3). In Creole and Garifuna women (15–59 years) HIV/AIDS and diabetes are the top two causes of death, accounting for 30 to 45% of all deaths respectively (data not shown). In Creole and Garifuna men HIV/AIDS, cardiovascular disease and cancers contribute particularly to the high rates of communicable and non-communicable diseases, together accounting for roughly 20 to 40% of all deaths. An important question, which cannot be answered from the available data, is how much of the difference in mortality between groups is related to differences in behavioural risk factors and how much to poorer access to health care and the management of risk factors like hypertension, diabetes, and HIV infection. Clearly, the poorer economic and social infrastructures that underlie the high rates of violence will also contribute to both increased levels of risk factors and poor access to effective health care.

### Limitations of This Study

The validity of our findings are dependent upon the completeness and timeliness of death registration, the completeness of the population census data, and the designation and accuracy of ethnicity at death and at the time of the census. Based on comparison to the number of deaths that would have been expected according to United Nations modelled estimates of mortality[23] we found no consistent evidence for an undercount in the figures we have used. Our findings are also robust to allocating to different ethnic groups the 5.8% of deaths that lacked ethnicity at registration. It is also worth noting that death registration in Belize is through its electronic health information system, meaning that it is updated almost immediately, and that our study was conducted over 3 years after the time period of interest: in other words, was based on the complete data available for the study period.

Our denominator population for 2010 was based on the census data provided by the Statistical Office of Belize, and is corrected for undercount. Missing data on ethnicity is minimal in the census data, at 0.29%. However, we do not know how consistently ethnicity is recorded between the census and at death registration, and whether what is recorded at the census might systematically differ to what is recorded at death registration. We have no reason to suspect systematic differences but it would require a new study to fully investigate this. Another potential source of bias, which would also require a different type of study to evaluate, is the possibility differential migration between ethnic groups of healthy and sick individuals. For example, it is possible that in some ethnic groups individuals working outside Belize might be more likely to return home to Belize when they become sick. While this seems possible for causes of death from chronic disease such as diabetes, cardiovascular diseases and cancers, it is much less plausible as a contribution to differences in death rates for injuries.

Finally it is important to acknowledge that the available data have not enabled us to dig beneath ‘ethnicity’, and to examine factors such as education, occupation, and income that will vary by ethnicity and contribute to differential mortality.

## Conclusion

We have found marked inequalities in adult mortality by ethnic group in Belize. Two ethnic groups have a mortality experience typical of parts of sub-Saharan Africa and the other two an experience common in parts of Western Europe and North America. Violent death contributes to this difference, particularly to the higher mortality in Creole men, where roughly 1 in 7 (based on our findings) can expect to die a violent death before their 60^th^ birthday. Communicable and non-communicable disease mortality also occurs at much higher rates in the Creole and Garifuna men and women compared to the Mayan and Mestizo. The Creole and Garifuna are the groups of largely African descent, and these findings likely reflect social and health inequities that have existed for centuries largely attributed to persistence of historical patterns in land ownership from days of slavery, ethnic discrimination, social status, living conditions and access to health care. Unfortunately, we have been unable to explore such socio-economic factors in these data, but hypothesize that they play a key role.

Our findings are a starting point for identifying health inequities in Belize. What must follow now is work to target potential interventions aimed at reducing the excess adult mortality in the Garifuna and Creole groups. Our findings are also a rare description from the Caribbean region of mortality by a social determinant, in this case ethnicity. We hope that other researchers will contribute analyses to understanding the social determinants of health in the Caribbean and the identification of health inequities. This work is essential if countries are to meet their commitments, made at the 2011 Rio Political Summit, to monitor, target, and reduce health inequities[32].

## Supporting Information

S1 Data Data to support the sensitivity analysis. (DOCX)

S1 File Census data. (XLSX)

## References

[1] United Nations DoEaSA, Population Division, Changing Levels and Trends in Mortality: the role of patterns of death by cause New York United Nations, 2012 Contract No.: ST/ESA/SER.A/318.

[2] United Nations DoEaSA, Population Division,. World Population Prospects: The 2012 Revision, CD Rom Edition. New York: United Nations, 2013 Contract No.: ESA/P/WP.228.

[3] Marmot M. Social determinants of health inequalities. Lancet (London, England). 2005;365(9464):1099–104. 10.1016/S0140-6736(05)71146-6 15781105

[4] World Health Organization. Closing the gap in generation: Health equality through action on the social determinants of health. Final Report of the Commission on Social Determinants of Health. Geneva: World Health Organisation, 2008.

[5] Government of Belize and the Caribbean Development Bank. Country Poverty Assessment Final Report: Volume 1. Main Report. London: Halcrow Group Limited, 2010.

[6] Community C. Member States and Associate Members Georgetown: CARICOM; 2016 [cited 2016 12 May]. Available from: http://caricom.org/about-caricom/who-we-are/our-governance/members-and-associate-members/.

[7] United Nations DoEaSA, Statistics Division,. World Statistics Pocket Book: Belize New York: United Nations; 2016 [cited 2016 12 May]. Available from: http://data.un.org/CountryProfile.aspx?crName=BELIZE.

[8] Statistical Institute of Belize. Abstract of Statistics: Belize Belize: Statistical Institute of Belize, 2012.

[9] Pan American Health Organization. Health in the Americas 2012 Edition: Regional Outlook and Country Profiles. Washington DC: PAHO, 2012.

[10] Mwakikagile G. Belize and Its Identity: A Multicultural Perspective New Africa Press; 2010.

[11] Institute for Health Metrics and Evaluation. GBD Profile: Belize: IHME GBD; 2010 [cited 2016 12 May]. Available from: http://www.healthdata.org/sites/default/files/files/country_profiles/GBD/ihme_gbd_country_report_belize.pdf.

[12] Feachem RGA, Phillips M, Kjellstrom T, Murray C, Over M. The Health of Adults in the Developing World: World Bank; 1992.

[13] Miller GJ, Kirkwood BR, Beckles GL, Alexis SD, Carson DC, Byam NT. Adult male all-cause, cardiovascular and cerebrovascular mortality in relation to ethnic group, systolic blood pressure and blood glucose concentration in Trinidad, West Indies. International journal of epidemiology. 1988;17(1):62–9. 3384551 10.1093/ije/17.1.62

[14] Thomas CN, Titus G, Williams D, Simeon D, Pitt-Miller P. Two-year mortality and its determinants following acute myocardial infarction in Trinidad and Tobago. The West Indian medical journal. 2000;49(2):112–4. 10948847

[15] Miller GJ, Cooper JA, Beckles GL. Cardiorespiratory fitness, all-cause mortality, and risk of cardiovascular disease in Trinidadian men—the St James survey. International journal of epidemiology. 2005;34(6):1387–94. 16169888 10.1093/ije/dyi193

[16] Ramsay-Johnson EM. An approach to reducing disparities in breast cancer in the United States Virgin Islands. The ABNF journal: official journal of the Association of Black Nursing Faculty in Higher Education, Inc. 2006;17(1):44–7. 16596900

[17] Odedina FT, Akinremi TO, Chinegwundoh F, Roberts R, Yu D, Reams RR, et al Prostate cancer disparities in Black men of African descent: a comparative literature review of prostate cancer burden among Black men in the United States, Caribbean, United Kingdom, and West Africa. Infectious agents and cancer. 2009;4 Suppl 1:S2 doi: 10.1186/1750-9378-4-S1-S2 19208207 10.1186/1750-9378-4-S1-S2 PMC2638461

[18] Ministry of Health GoB. Vital Registration in Belize: Memorandum of Understanding and Procedure Manual. Belize: Government of Belize, 2006.

[19] Epidemiology Unit MoH, Government of Belize,. Belize basic indicators 2010. Belize: Government of Belize, 2011.

[20] Organization PAH. Regional Mortality Database (updated March 2011): PAHO; 2011 [cited 2016 12 May]. Available from: http://www.paho.org/hq/index.php?option=com_content&view=article&id=4456%3A2010-download-detailed-data-files-paho-mortality-database&catid=2391%3Arho-databases&Itemid=2392&lang=en.

[21] Pan American Health Organization. Health situation in the Americas: basic indicators 2013. Washington DC: PAHO, 2013.

[22] Selvin S. Statistical Analysis of Epidemiologic Data. New York: Oxford University Press; 1991.

[23] United Nations DoEaSA, Population Division,. World Mortality Report 2011. New York: United Nations, 2012.

[24] United Nations. Model Life Tables for Developing Countries New York: United Nations, 1982 Contract No.: E.81.XIII.7.

[25] Fay MP, Feuer EJ. Confidence intervals for directly standardized rates: a method based on the gamma distribution. Statistics in medicine. 1997;16(7):791–801. 9131766 10.1002/(sici)1097-0258(19970415)16:7<791::aid-sim500>3.0.co;2-#

[26] Rajaratnam JK, Marcus JR, Levin-Rector A, Chalupka AN, Wang H, Dwyer L, et al Worldwide mortality in men and women aged 15–59 years from 1970 to 2010: a systematic analysis. Lancet (London, England). 2010;375(9727):1704–20. 10.1016/S0140-6736(10)60517-X 20434763

[27] Murray CJ, Kulkarni SC, Michaud C, Tomijima N, Bulzacchelli MT, Iandiorio TJ, et al Eight Americas: investigating mortality disparities across races, counties, and race-counties in the United States. PLoS medicine. 2006;3(9):e260 16968116 10.1371/journal.pmed.0030260 PMC1564165

[28] Crime UNOoDa. World Drug Report 2010 Vienna: United Nations, 2010 Contract No.: E.10.XI.13.

[29] Gayle H, Mortis N. Male Social Participation and Violence in Urban Belize: An Examination of Their Experience with Goals, Guns, Gangs, Gender, God, and Governance. Jamaica: University of the West Indies, Mona, Jamiaca & Ministry of Education, Government of Belize, 2010.

[30] Evans H. Gender differences in education in Jamaica. Kingston, Jamaica: Planning Institute of Jamaica and UNESCO, 1999.

[31] Chevannes B, Kingston:: Grace Kennedy Foundation. What We Sow and What We Reap: Violence and the Construction of Male Identity in Jamaica: Grace Kennedy Foundation Lecture Series 1999: Grace Kennedy; 1999 [cited 2016 12 May]. Available from: http://www.gracekennedy.com/images/lecture/GKF1999Lecture.pdf.

[32] World Health Organization Landmark conference ends with adoption of Rio Political Declaration Geneva: World Health Organization; 2012 [cited 2014]. Available from: http://www.who.int/sdhconference/en/.

