# Supplementary material for: Ethnic and Gender Disparities in Premature Adult Mortality in Belize 2008-2010
Source: PLoS One. 2016 Sep 19;11(9):e0163172. doi: 10.1371/journal.pone.0163172 (PMC5028024; doi:10.1371/journal.pone.0163172)
Supplement: S1 Data — (DOCX) [file pone.0163172.s001.docx]

**Supplementary Table and Figures**

**Table S1** Estimated completeness of mortality reporting in Belize between 1999 and 2011

| **COUNTRY** | **UN  PERIOD** | **ESTIMATED POPULATION ('000s)** | **UN ESTIMATED DEATHS** | **WHO  DATABASE DEATHS** | **ESTIMATED UNDERCOUNT (N)** | **ESTIMATED UNDERCOUNT (%)** | **AVERAGE UNDERCOUNT * (%)** |
| --- | --- | --- | --- | --- | --- | --- | --- |
| Belize | 1999 | 231.86 | 1290 | 1186 | 104 | 8.1 | -4.7 |
| Belize | 2000 | 238.586 | 1306 | 1534 | 0 | -17.5 | -1.6 |
| Belize | 2001 | 245.198 | 1319 | 1257 | 62 | 4.7 | -3.1 |
| Belize | 2002 | 251.766 | 1330 | 1284 | 46 | 3.5 | 4.3 |
| Belize | 2003 | 258.346 | 1339 | 1277 | 62 | 4.6 | 3.9 |
| Belize | 2004 | 265.04 | 1347 | 1298 | 49 | 3.6 | 2.4 |
| Belize | 2005 | 271.92 | 1356 | 1369 | 0 | -1 | 0.2 |
| Belize | 2006 | 278.985 | 1365 | 1395 | 0 | -2.2 | -1.3 |
| Belize | 2007 | 286.196 | 1376 | 1386 | 0 | -0.7 | 1.1 |
| Belize | 2008 | 293.544 | 1388 | 1301 | 87 | 6.3 | 0.6 |
| Belize | 2009 | 301.016 | 1402 | 1453 | 0 | -3.6 | -2.4 |
| Belize | 2010 | 308.595 | 1419 | 1557 | 0 | -9.7 | -7.2 |
| Belize | 2011 | 316.28 | 1438 | 1555 | 0 | -8.1 | -8.9 |

**Figure S1 Sensitivity Visualizations of the three major mortality groups for women and men: based on different levels of re-assignment of deaths with missing ethnicity data to each ethnic group in turn**

**COMMUNICABLE DISEASE (WOMEN)**

| Mayan |  |
| --- | --- |
| Creole |  |
| Garifuna |  |
| Mestizo |  |

**NCDs (WOMEN)**

| Mayan |  |
| --- | --- |
| Creole |  |
| Garifuna |  |
| Mestizo |  |

**INJURY (WOMEN)**

| Mayan |  |
| --- | --- |
| Creole |  |
| Garifuna |  |
| Mestizo |  |

**COMMUNICABLE DISEASE (MEN)**

| Mayan |  |
| --- | --- |
| Creole |  |
| Garifuna |  |
| Mestizo |  |

**NCDs (MEN)**

| Mayan |  |
| --- | --- |
| Creole |  |
| Garifuna |  |
| Mestizo |  |

**INJURY (MEN)**

| Mayan |  |
| --- | --- |
| Creole |  |
| Garifuna |  |
| Mestizo |  |
